# Recent Development of Fluoroquinolone Derivatives as Anticancer Agents

**DOI:** 10.3390/molecules29153538

**Published:** 2024-07-27

**Authors:** Justyna Nowakowska, Dominika Radomska, Robert Czarnomysy, Krzysztof Marciniec

**Affiliations:** 1 Department of Organic Chemistry, Medical University of Silesia, Jagiellonska 4, 41-200 Sosnowiec, Poland; 2 Department of Synthesis and Technology of Drugs, Medical University of Bialystok, Kilinskiego 1, 15-089 Bialystok, Poland; dominika.radomska@umb.edu.pl (D.R.); robert.czarnomysy@umb.edu.pl (R.C.)

**Keywords:** fluoroquinolones, anticancer, cytotoxic activity, metal ion complexes

## Abstract

Cancer is the second leading cause of death in the world following cardiovascular disease. Its treatment, including radiation therapy and surgical removal of the tumour, is based on pharmacotherapy, which prompts a constant search for new and more effective drugs. There are high costs associated with designing, synthesising, and marketing new substances. Drug repositioning is an attractive solution. Fluoroquinolones make up a group of synthetic antibiotics with a broad spectrum of activity in bacterial diseases. Moreover, those compounds are of particular interest to researchers as a result of reports of their antiproliferative effects on the cells of the most lethal cancers. This article presents the current progress in the development of new fluoroquinolone derivatives with potential anticancer and cytotoxic activity, as well as structure–activity relationships, along with possible directions for further development.

## 1. Introduction

The National Cancer Institute (NCI) defines cancer as a disease in which some of the body’s cells grow uncontrollably and spread throughout the body. Long periods of cell division, accumulation of oncogenic mutations, and favourable localisation of lesions allowing for the accumulation of abnormal cells appear to be necessary to initiate the carcinogenesis process [1]. According to statistical data, cancer is the second leading cause of death in the world following cardiovascular disease [2,3,4]. Among the most lethal cancers, the World Health Organisation indicates lung, colon, liver, stomach, and breast cancers [5]. Cancer treatment is based on a reductionist approach. The methods used primarily encompass pharmacotherapy (chemotherapy, immunotherapy), radiotherapy, and surgical removal of the lesion [6]. Despite medical progress, the prognosis for patients may remain unfavourable. The lack of a perfect panacea and the high costs of designing, synthesising, and marketing new medicinal substances encourage the search for an anticancer drug among derivatives of available drugs through the so-called repositioning of the drug [7].

Fluoroquinolones make up a group of synthetic antibacterial drugs with a broad spectrum of activity in invasive medical and veterinary infections. They are used in infections of the urogenital tract, respiratory tract, and gastrointestinal tract caused by Gram-positive and Gram-negative microorganisms [8]. They are also successfully used in the treatment of bacterial infections of the eyes, bones, joints, and soft tissues [7,8,9]. They also have antiviral activity [9,10]. Chemically, fluoroquinolones are derivatives created by modifying the skeleton of 4-oxo-1,4-dihydroquinolones. In addition to the oxygen groups necessary for pharmacological activity, namely, carboxyl and ketone groups at positions 3 and 4 of the ring skeleton, respectively, they contain one or two fluorine atoms in their structure [11,12].

Nalidixic acid obtained as a byproduct of the synthesis of chloroquine has been the first compound classified as a quinolone antibiotic. The ring structure of that compound is 1,8-naphthyridine [13]. Nalidixic acid was a drug with a narrow spectrum of pharmacological action widely used in the 1960s in urinary tract infections. Modifications of the structure of nalidixic acid have led to the preparation of approximately 10,000 analogues classified into four generations of quinolones [13,14]. In addition to nalidixic acid, representatives of the first generation of quinolones also include piromidic acid and pipemidic acid. Replacing the carbon atom with a nitrogen atom at position 6 of the nalidixic acid ring skeleton and introducing heterocyclic substituents at position 7 (in piromidic acid, the substituent is pyrrolidine with one nitrogen heteroatom, and in pipemidic acid, piperazine containing two nitrogen heteroatoms) has resulted in the preparation of drugs with an extended spectrum of activity against Gram-negative bacteria and that are very well absorbed after oral administration [15,16]. The second and third generations include 4-quinolone derivatives containing one or two halogen atoms in the heterocyclic system (most often fluorine–fluoroquinolones), while the fourth-generation antibiotics differ mainly in terms of modifications within the heterocyclic substituent at position 7. Those structural changes have a positive impact on the antibacterial activity properties of quinolones, extending the spectrum of their action onto Gram-positive bacteria, as well as anaerobic and atypical bacteria [11]. The most important representatives of the second-generation quinolones are ciprofloxacin, norfloxacin, ofloxacin, pefloxacin, lomefloxacin, and fenoxacin. The third generation includes levofloxacin, sparfloxacin, and grepafloxacin. The fourth-generation drugs include moxifloxacin and gemifloxacin.

The mechanism of the antibacterial action of fluoroquinolones is based on the inhibition of bacterial enzymes involved in DNA replication. By inhibiting DNA gyrase, they prevent the binding of initiation proteins, thus inhibiting the initiation of the replication process. Inhibition of topoisomerase IV prevents decatenation, thereby preventing the separation of genetic material into two daughter cells. Inhibition of proper DNA replication leads to apoptosis of bacterial cells [17]. Moreover, fluoroquinolones have anticancer potential, manifested by their ability to inhibit the cell cycle and induce apoptosis of cancer cells. In this review, we have decided to elaborate upon the current progress in the development of new fluoroquinolone derivatives showing anticancer properties and correlations between the structure of those compounds and their cytotoxic activity towards cancer cells. 

## 2. Structure of Fluoroquinolones and Their Antiproliferative Activity

The chemical structure of fluoroquinolones is very diverse, and small structural modifications may significantly affect their biological activity. Several structural elements of fluoroquinolones appear to be of particular importance in their mechanism of anticancer action.

**Figure 1:**
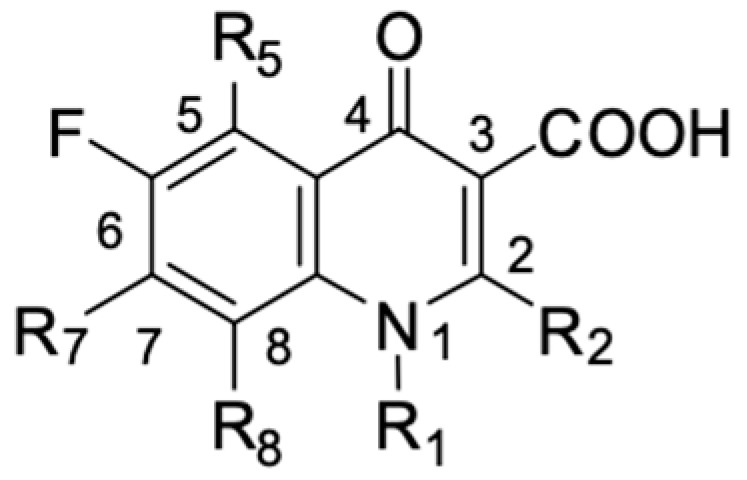
Structure of fluoroquinolones.

As mentioned below, the core of fluoroquinolones is a modified quinoline ring (Figure 1). Its structure is crucial in inhibiting topoisomerase II, which is an enzyme involved in numerous processes related to the replication of genetic material [18,19]. The nitrogen at position 1 is necessary for activity, and the type of substituent on this atom determines the potency of the activity. The related literature data indicate the advantage of the cyclic substituent (cyclopropyl) over chain substituents in the inhibitory effect on topoisomerase II [20]. In order to achieve anticancer activity, a fluorine atom at position 6 of the quinolone ring is necessary. The introduction of an additional fluorine atom or a methoxy group at position 8 increases the effectiveness of that action [18].

Similarly to the carboxyl group at position 3, the heterocyclic substituent at position 7 has a significant impact on the antibacterial activity of fluoroquinolones and the interaction with the prokaryotic topoisomerase-DNA complex, but it does not have an affinity for eukaryotic topoisomerases. It thus gives fluoroquinolones selectivity towards bacterial enzymes. In order to change the profile of action toward cancer cells, it is important to induce modifications within both functional groups [18,20,21].

Based on this information, five representatives of fluoroquinolones were selected, and their tumorigenic activity against cancer cell lines was characterised. The data are summarised in Table 1, along with the structural formulas of the selected compounds.

## 3. Complexes of Fluoroquinolones with Heavy Metals

Among cytostatic drugs currently used in cancer pharmacotherapy, platinum-based drugs such as cisplatin, carboplatin, and oxaliplatin, which are coordination compounds of Pt^2+^ ions, are widely used. Platinum complexes interact with the DNA of cancer cells, disrupting the structure of the DNA and interfering with its synthesis. They inhibit cell division, which results in the induction of apoptosis. They are used within the framework of monotherapy or in combination with other anticancer drugs for the treatment of breast, ovarian, or colorectal cancer [37,38,39,40,41].

**Figure 2:**
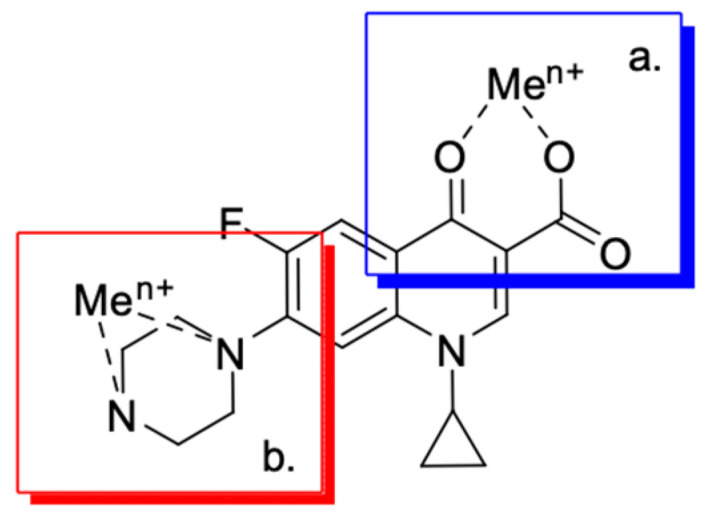
Scheme of metal ion chelation exemplified by the structure of ciprofloxacin, showing the formation of *O,O′* coordination (**a**) and *N,N′* coordination (**b**).

Fluoroquinolones are able to form complex compounds through the coordination of *O,O′*-bidentate or through coordination through nitrogen atoms in the piperazine *N,N′* ring, so they constitute a chelate ligand of bidentate (Figure 2). The formation of a complex involving the carbonyl and carboxyl oxygen atoms at positions 3 and 4 of the quinolone skeleton in fluoroquinolones is associated with obtaining derivatives that exhibit a wide spectrum of biological activity, including higher antibacterial and cytotoxic potential compared to the original ligand. Chelation of metal ions by piperazine nitrogen atoms is a less common phenomenon, but it may result in derivatives with antiproliferative activity [42]. The biological activity and stability of metal–fluoroquinolone complexes vary and depend on both the type of bidentate and the ligand (Table 2).

Knowing that platinum compounds are used successfully in the treatment of cancer, it seems beneficial to use this metal and other heavy metals in combination with fluoroquinolones. The synthesis of platinum–fluoroquinolone complexes using ciprofloxacin, levofloxacin, sparfloxacin, gatifloxacin, and ofloxacin occurs through coordination in the piperazine ring [43]. The results of the cytotoxicity tests of the complex with sparfloxacin as the ligand indicate stronger inhibitory activity against breast cancer cells and greater selectivity as compared to cisplatin [44]. The same coordination is demonstrated by gold(III) complexes with norfloxacin, levofloxacin, or sparfloxacin. They exhibit similar cytotoxic activity against lymphoma, myeloid leukaemia, and melanoma cell lines: they inhibit the cell cycle in the G0/G1 phase and induce apoptosis in a concentration-dependent manner [45]. Replacing platinum(II) or gold(II) with another metal ion causes a change in coordination with the ligand from *N,N′* to *O,O′*. Among the coordination compounds of fluoroquinolones with semiprecious and noble metals, rhenium complexes are also known. Rhenium(I) in coordination with enrofloxacin shows stronger inhibition of DNA topoisomerase as compared to the ligand alone [46]. The results of cytotoxicity tests of complexes with enrofloxacin and levofloxacin indicate their activity against erythroleukaemia cells [47].

**Table 2:** Activity of fluoroquinolone complexes.

	The Structure of the Complex	Cell Line or DNA/Activity	Ref.
**6**	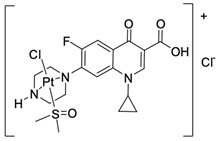	MCF-7/17.3 µM MDA-MB-231/15.3 µM	[42]
**7**	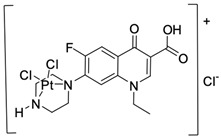	BF16-F10/27 µM	[43]
**8**	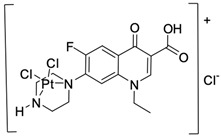	BF16-F10/29 µM	[43]
**9**	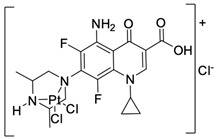	BF16-F10/45 µM	[43]
**10**	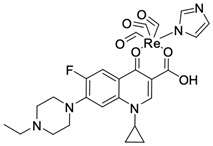	K-562/13.12 µM	[44,45]
**11**	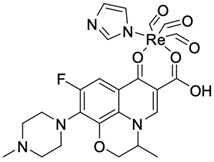	K-562/19.99 µM	[45]
**12**	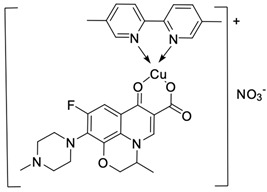	A498/GI_50_ < 10	[46]
**13**	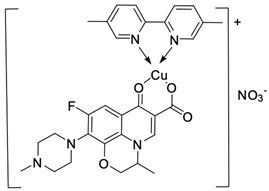	MCF-7/1.04 µM	[48]
**14**	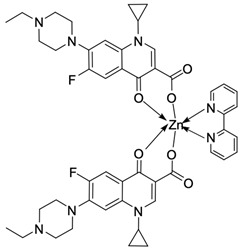	Ct DNA/Kb = 2.61 × 10^6^ M^−1^	[49]
**15**	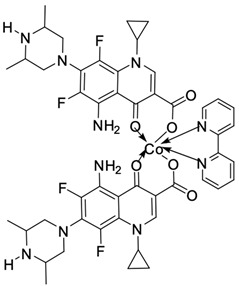	Ct DNA/Kb = 1.38 × 10^6^ M^−1^	[50]
**16**	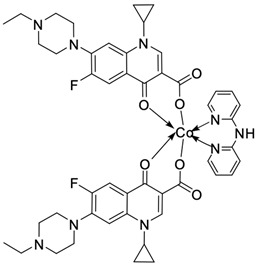	Ct DNA/Kb = 3.39 × 10^6^ M^−1^	[51]
**17**	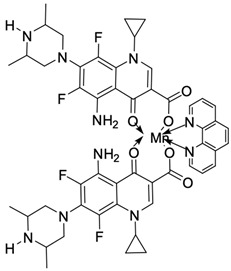	Ct DNA/Kb = 1.01 × 10^6^ M^−1^	[52]
**18**	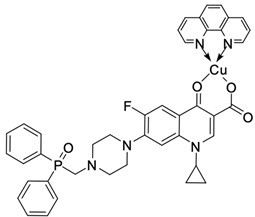	A549/8.3 µM CT26/7.3 µM	[53]
**19**	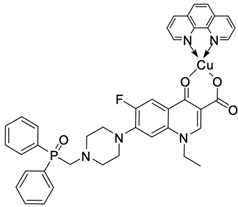	A549/15.9 µM CT26/8.4 µM	[53]
**20**	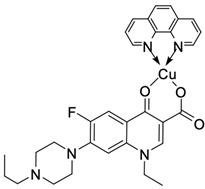	HL-60/induces apoptosis in 57.59% at concentration 100 µg × mL^−1^	[54]
**21**	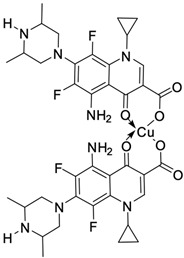	BT20/12 µM	[55]
**22**	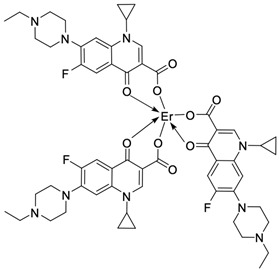	Ct DNA/Kb = 4.24 × 10^6^ M^−1^	[56]

Among the transition metals, fluoroquinolones form complexes with similar or higher biological activity as compared to the free ligand with ions such as Cu(I) [48,57,58]; Cu(II) [49,50,51,52,53,54,55,56,58,59,60,61,62]; Zn(II) [63,64,65,66,67]; Co(II) [68,69,70,71,72]; or Mn(II) [73]. Copper(II) coordination compounds are particularly interesting because of their broad spectrum of antiproliferative activity and much lower toxicity toward healthy cells than the platinum compounds currently used in chemotherapy. The chelation of copper ions by ciprofloxacin results in compounds that increase the production of reactive oxygen species and induce apoptosis in human lung adenocarcinoma cells. Similar effects are achieved by using norfloxacin as the ligand [57]. Furthermore, copper(II) norfloxacin complexes prove antiproliferative effects on osteosarcoma and myeloid leukaemia cells [74,75], and changing the ligand into sparfloxacin improves the inhibition of hormone-independent breast cancer cells [76]. The use of pefloxacin as a ligand leads to the production of active copper complexes against breast cancer cells, and that activity exceeds the activity of analogous nickel(II) complexes [77]. Copper(II) complexes based on pefloxacin and statin derivatives effectively inhibit the proliferation of human colorectal cancer cells, reduce the viability and clonogenic capacity of cells, and induce apoptosis [78].

Lanthanide complexes are becoming increasingly important in cancer diagnosis and therapy. They are used, among others, as contrast agents in magnetic resonance imaging and in cancer radiotherapy [79]. The terbium(III)–ciprofloxacin complex, characterised by green fluorescence, may be successfully used as a fluorescent probe [80]. Lanthanide compounds with fluoroquinolone ligands, namely, ciprofloxacin [81,82], enrofloxacin [83], gemifloxacin [66,84] levofloxacin [85], norfloxacin [86], and sparfloxacin [87], show high antimicrobial activity. The interest in erbium(III) complexes with fluoroquinolones has led to the preparation of compounds with cytotoxic and antimicrobial activity that exceeds the activity of other metal(II)–quinolone complexes found in the related literature [88]. Those reports may constitute a strong foundation for further attempts to obtain new lanthanide complexes and study their cytotoxicity toward cancer cell lines.

## 4. Fluoroquinolone Derivatives and Their Anticancer Properties

**Figure 3:**
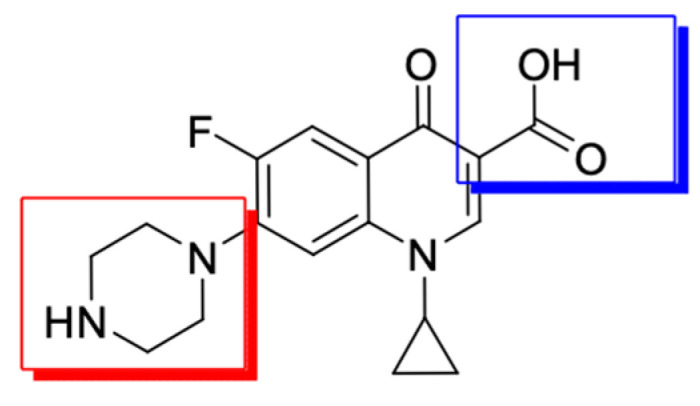
The main modification sites of the fluoroquinolone molecule exemplified by ciprofloxacin (modifications in position 7 are marked in red and modifications in position 3 are marked in blue).

It is possible to modify the structure of a fluoroquinolone in many directions through substitutions at the 1 and 6 positions, modification of substituents at C3 and C7, and the addition of another fluorine atom at the 8 position. Modifications in the quinolone core led to changes in the antimicrobial activity [89]. The attachment of substituents [70] (Figure 3) is associated with the spectrum expansion of drug activity beyond antibacterial, to antiviral [90,91] and anticancer.

Substitution of the 7 position and substitution of the *N*-4-piperazinyl moiety have a large impact on the potency, bioavailability, and physicochemical properties [18,92]. As a result of the synthetic work of researchers, there is more and more information in the related literature about new fluoroquinolone derivatives that show anticancer activity in relation to human cell lines of the deadliest cancers.

**Figure 4:**
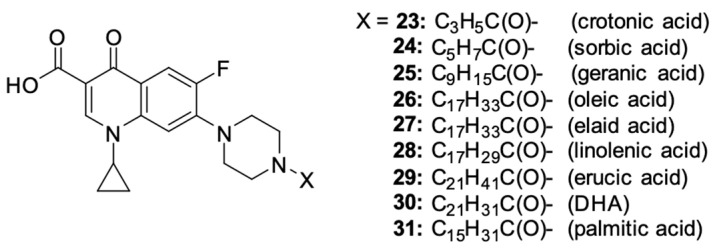
Structure of ciprofloxacin amide derivatives obtained by conjugation with fatty acids.

The latest literature reports expand the horizons in terms of the directions for obtaining derivatives. Chrzanowska et al. [93] obtained various amide derivatives of ciprofloxacin as a result of the condensation of a fluoroquinolone with fatty acids (Figure 4). The selection of acids with varied chain lengths, degrees of unsaturation, and geometric isomerism ensured the structural variability of the compounds obtained. The synthetic route used crotonic (**23**), sorbic (**24**), geranium (**25**), oleic (**26**), elanic (**27**), linolenic (**28**), erucic (**29**), DHA (**30**)**,** and palmitic (**31**) acids. The efficiency of the syntheses ranged from 44% to 59%. Biological tests allowed for the assessment of the cytotoxicity and apoptosis-inducing effect in primary and metastatic SW480 and SW620 colon cancer cells and in PC3 prostate cancer cells.

The strongest antiproliferative properties were found in PC3 cells for derivatives **24**, **26**, and **27**, as compared to unsubstituted ciprofloxacin. The IC_50_ values were 11.7, 7.7 and 15.3 µM, respectively. Conjugates **28** and **30** showed moderate activity (34.4 and 27.8 µM, respectively). The most promising inhibitory effect on the SW480 cell line was demonstrated by conjugates **24**, **25**, **27**, and **30** conjugated with polyunsaturated fatty acids, with IC_50_ values ranging between 20.1 and 35.7 µM. The obtained ciprofloxacin amides showed high selectivity toward cancer cells, proving no cytotoxic effect towards normal human HaCaT keratinocytes. Furthermore, derivatives **24**, **26**, **27**, and **30** showed noticeable apoptosis-inducing properties in the selected cell lines. These compounds significantly influenced the increase in the number of cells in the late phase of apoptosis at concentrations ranging from 600 to 1500 μM, and the ciprofloxacin–oleic acid conjugate had the highest pro-apoptotic ability among those mentioned.

**Figure 5:**
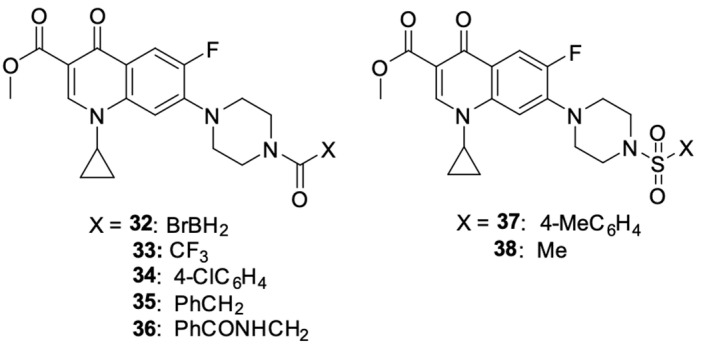
*N*-acylated and *N*-sulfonated derivatives of ciprofloxacin.

Akhtar et al. [94] modified the structure of ciprofloxacin in two ways: esterifying the carboxyl group at position C3 and attaching a substituent to the nitrogen in the piperazine ring at position 7. The synthetic work gave rise to *N*-acylated derivatives **32**–**38** (Figure 5).

The obtained derivatives were tested for cytotoxic activity against the MCF-7 breast cancer cell line using ciprofloxacin as the standard drug. All the compounds showed higher anticancer activity than unsubstituted ciprofloxacin. The highest cytotoxic potential was demonstrated by compounds **32** (IC_50_ = 4.3 µM), **33** (IC_50_ = 12.9 µM), and **35** (IC_50_ = 60.9 µM), among which derivative **32** was singled out, and its usefulness in future syntheses of new *N*-alkylated fluoroquinolone derivatives with improved anticancer properties was indicated. The mechanism of the anticancer activity of compound **32** was investigated using in silico modelling methods. The results indicated topoisomerase II as a possible cytotoxic target; moreover, this derivative showed higher affinity for this enzyme than the reference unsubstituted ciprofloxacin.

**Figure 6:**
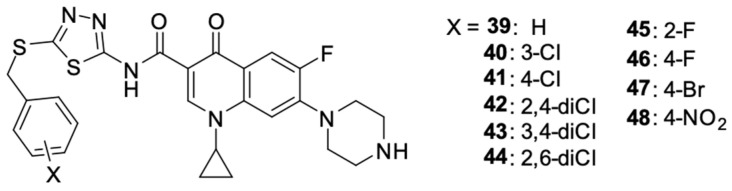
Ciprofloxacin derivatives containing *N*-(5-(benzylthio)-1,3,4-thiadiazol-2-yl)carboxamide moiety.

The syntheses conducted by Ahadi et al. [95] led to the preparation of a series of ciprofloxacin derivatives **39**–**43** (Figure 6). Modifications of the fluoroquinolone structure resulted in *N*-(5-(benzylthio)-1,3,4-tiadazol-2-yl)carboxamide moiety at position 3.

The synthesised compounds were assessed for their activity against selected human cancer cell lines: MCF-7 breast cancer, A549 lung cancer, and SKOV-3 ovarian cancer. Derivatives **39**–**48** showed anticancer activity against each of the selected cell lines and were characterised by the stronger antiproliferative activity against MCF-7 cells than A549 and SKOV-3. The cytotoxic activity against the tested cell lines expressed in IC_50_ values for the derivatives is summarized in Table 3.

The introduction of fluorine into the benzyl group in compounds **45** and **46** resulted in favourable changes in the activity against SKOV-3, and the bromine-containing derivative **47** showed higher activity against A549 cells. The nitro group in the para position of the benzyl group reduced the activity of that compound against each of the selected cancer cell lines. The mechanism of the anticancer action of the derivatives was based on the inhibition of the cell cycle in sub-G1 phase and the induction of oligonucleosomal DNA fragmentation. Compounds **43** and **45** showed a concentration-dependent pro-apoptotic effect.

**Figure 7:**
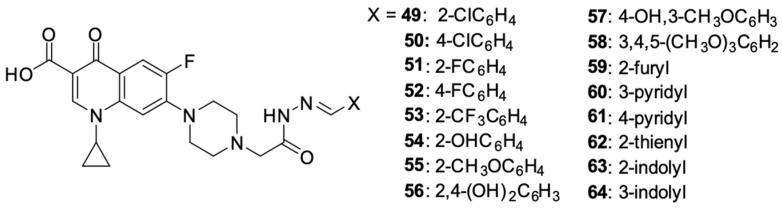
Ciprofloxacin derivatives containing topoisomerase II inhibitor structural elements.

Kassab et al. [96] prepared a series of derivatives **49**–**70** with structural features of a topoisomerase II inhibitor, using ciprofloxacin substituted at position 4 of the piperazine ring with *N*-acetylarylhydrazone, oxadiazole, or its bioisosterotriazole scaffolds. Aryl and heteroaryl groups were introduced into the *N*-acetylarylhydrazone residue. The structures of the synthesised derivatives are shown in Figure 7.

An assessment of the anticancer activity performed on 59 panels of human cancer cell lines showed strong activity of derivatives **53** against UO-31 (IC_50_ = 4.92 μM) and MDA-MB-468 (IC_50_ = 2.16 μM) cell lines, **54** and **56** relative to the U0-31 cell line (IC_50_ 0.75 μM and 3.19 μM, respectively), **60** relative to NCI-H226 cells (IC_50_ = 1.02 μM), IGROV1 (IC_50_ = 0.75 μM) and UO-31 (IC_50_ = 0.72 μM), and **63** relative to the HL60 cell line (IC_50_ = 1.55 μM). The antiproliferative activity of the obtained compounds seemed to correlate effectively with their ability to inhibit topoisomerase II. The highest ability to inhibit that enzyme was demonstrated by derivatives **54** and **63**, which was several times higher than the activity of the reference compounds: doxorubicin, amsacrine, and etoposide. The antiproliferative effect of the derivatives led to the inhibition of the cell cycle in the G2/M phase and the induction of the intrinsic mitochondrial apoptosis pathway.

**Figure 8:**
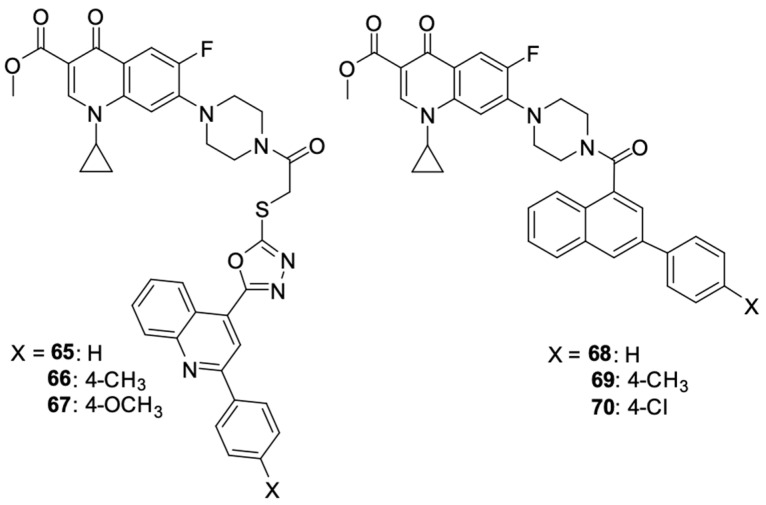
Structure of amidopiperazine derivatives of ciprofloxacin.

Ezelarab et al. [97] obtained a series of ciprofloxacin and quinoline derivatives **65**–**70** as a result of a multi-stage synthetic process (Figure 8). Those hybrids were assessed for their anticancer properties against a range of cell lines. Studies have shown significant cytotoxic activity of derivatives **65** and **66** against SR-leukaemia and UO-31 renal cell carcinoma cell lines. The antiproliferative activity was expressed as a percentage of growth inhibition. Derivatives **65** and **66** inhibited the growth of 33.25 and 52.62% of leukaemia cells, respectively, and 64.19 and 55.49% of renal cancer cells, respectively. Moreover, they inhibited the growth of LOX IMVI melanoma cells by 39.14 and 36.64%, respectively.

The studies showed the significant cytotoxic activity of derivatives **65**, **66**, and **70** against the selected cell lines. These compounds inhibited the cell cycle and induced apoptosis of the cancer cells, thereby reducing their growth and viability. In addition to their antiproliferative properties, ciprofloxacin/quinoline derivatives demonstrated potent antifungal activity against Candida species, highlighting their potential to be multi-targeted therapeutic agents.

**Figure 9:**
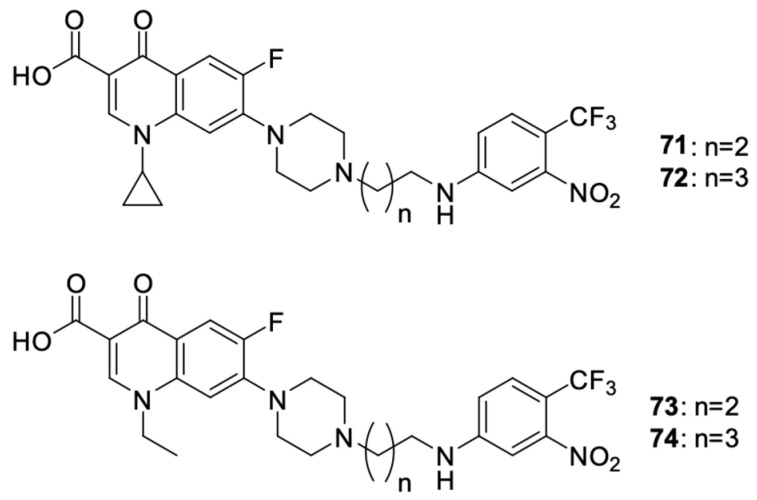
Photodonor hybrids of ciprofloxacin and norfloxacin.

Fallica et al. [98] presented the synthetic path and results of the research on the antiproliferative effect of photodonor hybrids of nitric oxide (NO) with fluoroquinolones ciprofloxacin and norfloxacin (Figure 9). The synthesis involved modification of the fluoroquinolone structure with NO donor moieties, creating compounds that released NO upon exposure to light. Their antiproliferative effect was tested on the DU145 and PC3 prostate cancer, HCT116 colon cancer, and MDA-MB231 and MCF-7 breast cancer cell lines. In vitro experiments indicated that derivatives **71**–**74** were effective against the selected cell lines. The effect of the norfloxacin derivatives was stronger than that of the ciprofloxacin derivatives. The best results were achieved with norfloxacin derivative **73**: the IC_50_ values for the PC3, MCF7, and MDA-MB231 cell lines were 2.33 μM, 2.27 μM, and 1.52 μM, respectively. In relation to the DU145 cell line, derivative **74** was the most effective (IC_50_ = 1.56 μM). Cytotoxicity studies showed that regardless of the length of the carbon bridge, the carboxyl moiety was indispensable for the anticancer effect. At the same time, it was observed that these compounds exhibit similar cytotoxicity towards non-cancer cell lines HBL100 and WH1. The IC_50_ values ranged from 3.51 to 13.2 μM depending on the derivative and the cell line tested. Similarly, the norfloxacin derivatives showed higher cytotoxicity.

**Figure 10:**
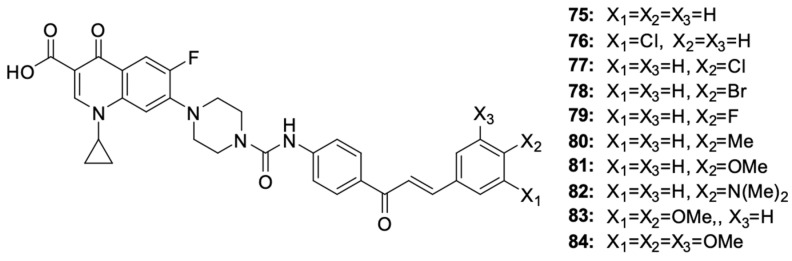
Chalcone derivatives of ciprofloxacin.

Hihh et al. [99] modified the ciprofloxacin molecule by combining various derivatives through a urea linker and a piperazine ring at position 7 of the fluoroquinolone (Figure 10). The cytotoxic activity of new hybrids **75**–**84** was assessed, among others, against the HCT-116 colon cancer and leukaemia-SR cell lines.

Among the chalcone derivatives of ciprofloxacin, compounds **77** and **84** showed strong growth inhibitory effects on HCT-116 cells (IC_50_ values of 2.53 μM and 2.01 μM, respectively) and leukaemia-SR cells (IC_50_ values of 0.73 μM and 0.63 μM, respectively). Additionally, they showed a significant inhibitory activity against topoisomerase, comparable to the activity of the reference compounds camptothecin and topotecan. Cytotoxicity studies of the derivatives indicated a stronger toxic effect on leukaemia cells than on colon cancer cells. Compounds **76** and **81** showed very good growth inhibitory effects on leukaemia cells, with IC_50_ of 2.38 μM and 3.22 μM, respectively. Changing the position of the chlorine atom from 4 to 3 in compound **76** or replacing it with other halogens at position 4 in the case of derivatives **78** and **79** reduced the cytotoxic effect. Studies on the impact of compounds on the cell cycle progression indicated hybrid **84** to be the substance that inhibited the cell cycle in the G2/M phase and apoptosis in leukaemia cells. Testing the cytotoxicity of compounds **77** and **84** towards the non-cancerous WI-38 cell line indicated the selectivity of the substance towards cancer cells. The IC_50_ values for the WI-38 cells were 15.96 μM and 18.42 μM, respectively, being 6 to even 20 times higher than the IC_50_ values determined for cancer cells.

**Figure 11:**
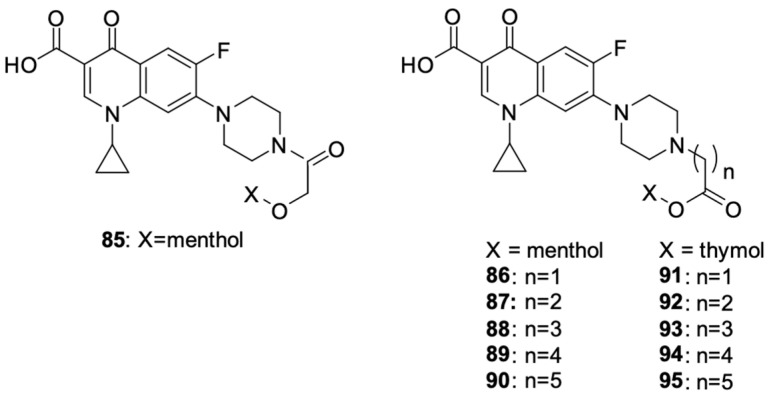
Menthol and thymol derivatives of ciprofloxacin.

Szostek et al. [100] designed and synthesised ciprofloxacin derivatives using menthol and thymol moieties attached to the fluoroquinolone using various carboxyl linkers (Figure 11). Both *N* and *O*-derivatives were obtained, as well as di compounds substituted at position carboxyl group in the 3 position and at the nitrogen atom of the piperazine ring in position 7, but the assessment of the biological activity of the latter did not yield satisfactory results. The best antiproliferative activity against HepG2 liver cancer cells, HCT-116 colon cancer cells, and SW480 and SW620 colon cancer cells was demonstrated by derivative **87**, with IC_50_ values against the mentioned lines of 36.8, 24.2, 30.3 and 38.6 μM, respectively; derivative **92**, with IC_50_ values of 51.3, 39.1,33.7 and 43.5 μM, respectively; and derivative **95**, with IC_50_ values of 41.8, 30.5, 29.5 and 49.6 μM, respectively, while being non-toxic towards non-cancer HaCaT cells (IC_50_ = 45.5, >100 and 64.9 μM for derivatives **87**, **92**, and **95**, respectively).

**Figure 12:**
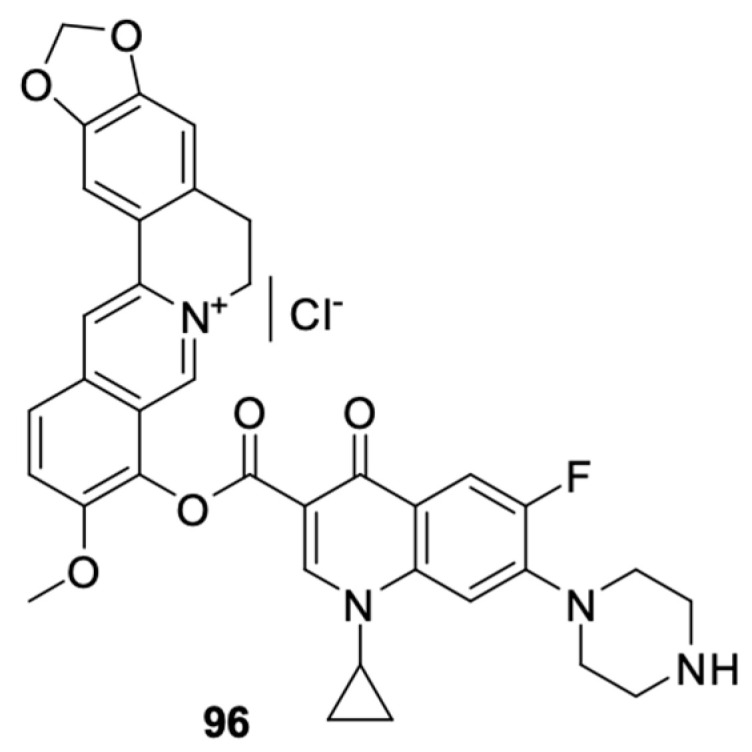
Conjugate of berberine and ciprofloxacin.

Results proving the beneficial effects of substances of natural origin, such as curcumin, silybin, or berberine, on the inhibition of the development of cancer cells and their potential use in therapy have recently appeared more and more frequently [101,102,103,104,105,106,107,108]. Milata et al. [109] synthesised a number of 9-*O*-substituted berberine derivatives, including the condensation product of berberine with ciprofloxacin (Figure 12). The anticancer effects of the compounds were tested on HeLa and HL-60 cell lines. Derivative **96** (mean IC_50_ = 19.3 µM) showed greater antiproliferative activity towards HL-60 cells than the non-substituted berberine and ciprofloxacin but had a weaker effect than the other compounds. That hybrid was found to have inhibited the cell cycle in the HL-60 leukaemia cell line in the G2/M phase.

A well-known modification of fluoroquinolones to obtain anticancer compounds is the formation of Mannich bases. The ortho-phenol chalcone derivative of ciprofloxacin **97** obtained by Alaaeldin et al. [110] showed beneficial properties. It inhibited topoisomerase I and II and had antiproliferative effects on A549 lung cancer (IC_50_ = 27.71 µM) and HepG2 hepatoma cells (IC_50_ = 22.09 µM). Furthermore, it inhibited the cell cycle in the G2/M phase and activated the apoptotic pathway. Additionally, exposure of non-cancer cells to derivative **97** indicated high selectivity of the cytotoxic effect of the substance towards cancer cells. The IC50 value for the WI38 cell line was 118.65 μM.

**Figure 13:**
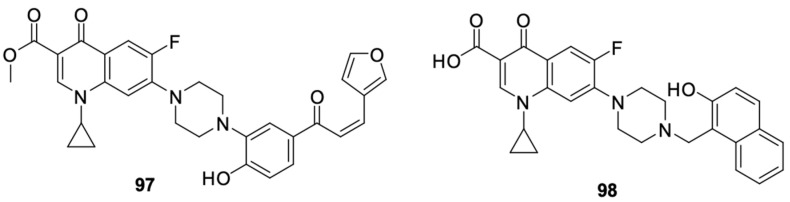
Ortho-phenol Mannich bases derived from ciprofloxacin.

Fawzy et al. [111] synthesised and tested naphthol Mannich base **98** for anticancer activity (Figure 13). The compound showed antiproliferative activity against the OVCAR-3 ovarian cancer and A-549 lung cancer cell lines by inhibiting the cell cycle in the S phase and inducing apoptosis through the mitochondrial pathway apoptotic.

**Figure 14:**
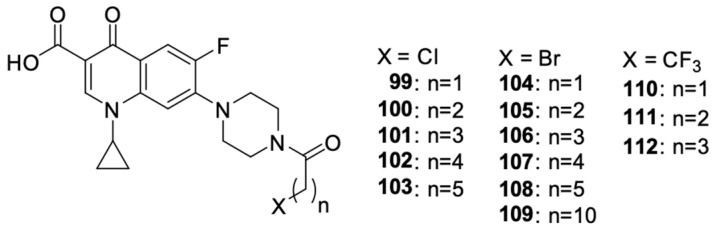
*N*-acylated ciprofloxacin derivatives.

Struga et al. [112] obtained a series of *N*-acylated ciprofloxacin derivatives (Figure 14) and conjugates of ciprofloxacin molecules connected with carbon linkers of various lengths. The antiproliferative activity of compounds **99**–**112** was proven against PC3 prostate cancer cells. Derivative **99** showed high antiproliferative activity against the selected cell line, much stronger than in the case of cisplatin. For this compound, the IC_50_ value was 2.02 μM. Furthermore, it induced apoptosis/necrosis in prostate cancer cells.

**Figure 15:**
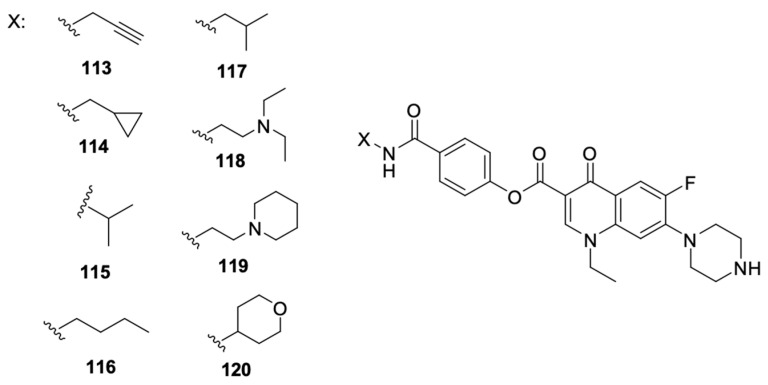
Benzamide derivatives of norfloxacin.

Recent literature reports also include data on norfloxacin and levofloxacin derivatives, in addition to ciprofloxacin derivatives. Xi et al. [113] focused on obtaining inhibitors of microRNA-21, which is overexpressed in cancer cells. They designed and synthesised a series of benzamide derivatives of norfloxacin **113**–**120** (Figure 15).

Derivative **119** showed the best efficacy as a microRNA-21 inhibitor, and that effect was comparable to the selected small molecule inhibitor. That compound suppressed the expression at the level of transcription of its original form. Evaluation of the antiproliferative activity against HCT-116 and HeLa cell lines confirmed the ability to inhibit colony formation and migration and induction of apoptosis in the case of compound **119.** That work was a continuation of the research conducted and described by Hei et al. [114], who indicated norfloxacin derivatives among the compounds of other fluoroquinolones, namely, ciprofloxacin, levofloxacin, gatifloxacin, and enoxacin, to be potential microRNA-21 inhibitors.

**Figure 16:**
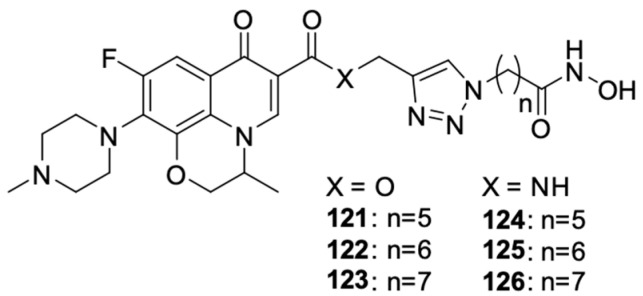
Levofloxacin and hydroxamic acid derivatives.

Wang et al. [115] linked levofloxacin with hydroxamic acid using carbon linkers of varied lengths, resulting in dual-acting derivatives targeting histone deacetylase and tubulin polymerisation (Figure 16). Modification of the length of the carbon chain had a significant impact on the inhibitory activity of the compound. The antiproliferative properties of the new hybrids were tested on A549 cell lines HepG2, MCF-7, PC-3, and HeLa. Compound **125** had the best anticancer properties and was more than 20 times stronger than free levofloxacin. The IC_50_ values of the derivative against these cell lines were 2.1 μM, 2.3 μM, 0.3 μM, 4.9 μM, and 1.1 μM, respectively, while the IC_50_ values of levofloxacin ranged from 64.2 to a value exceeding 100 μM in relation to individual cell lines.

To sum up, the examples described above show interesting directions of work leading to the preparation of anticancer compounds based on the fluoroquinolone skeleton. N-substituted compounds have higher cytotoxic potency compared to C-substituted derivatives, and the most frequently chosen substrate is ciprofloxacin. Nevertheless, these reports may constitute the basis for the syntheses of analogous derivatives using other fluoroquinolones in order to expand the range of potential therapeutics.

## 5. Molecular Docking

The search for new medicinal compounds is becoming increasingly difficult. This is associated with increasing research costs and stricter experimental procedures. In this context, modern computational methods are helpful, as they enable a large part of preliminary research to be carried out in silico. One of these methods is molecular docking, which has become a powerful tool for drug development (including the repositioning of known drugs) and is constantly evolving. Docking is a method that allows for determining the distribution and conformation of a ligand at the receptor binding site. It also enables the assessment of the binding strength of the complex. It is, of course, used in in silico research on the anticancer activity of fluoroquinolones and their derivatives. 

Mcl-1 is a potent anti-apoptotic protein that is amplified in many human cancers, while the microphthalmia-related transcription factor (MITF) promotes cell proliferation and plays a pro-survival role. Beberok et al. conducted in silico studies on the possible interaction of ciprofloxacin with MITF/Mcl-1 proteins. The analysis showed that ciprofloxacin has the ability to form complexes with MITF and Mcl-1 proteins and may thus have an apoptotic effect [116].

**Figure 17:**
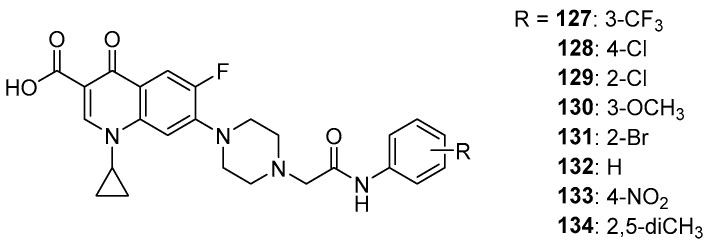
Ciprofloxacin amide derivatives.

Suresh et. al. performed docking of a series of ciprofloxacin derivatives to topoisomerase II (Figure 17) using the Glide program. These derivatives obtained docking score values ranging from −7.78 to −8.04 kcal/mol and were lower than the value obtained for the reference ciprofloxacin (−7.57 kcal/mol), indicating higher stability of the obtained derivative complexes **127**–**134** than the reference [117].

**Figure 18:**
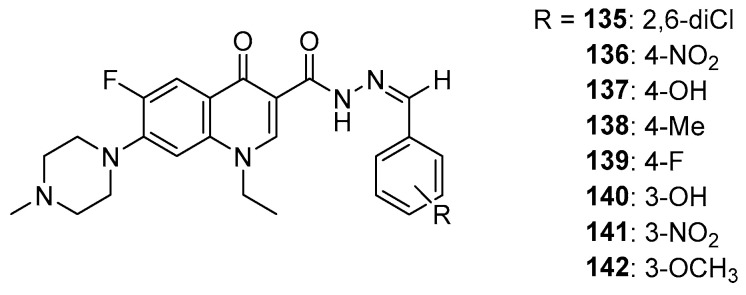
Pefloxacin hydrazones.

In silico studies by Allaka et al. also used topoisomerase II as a protein target. A series of hydrazone derivatives of pefloxacin were used as ligands (Figure 18). The obtained docking score values for hydrazones **135**–**142** ranged from −7.29 kcal/mol to −6.27 kcal/mol, and for the reference compound, this value was −5.80 kcal/mol [118]. This result may indicate the potential anticancer activity of the tested derivatives. 

## 6. Methods

A literature review of reviews and studies was performed from 2000 to 2024. Using terms such as ‘anticancer drugs’, ‘fluoroquinolone’, and ‘fluoroquinolones derivatives’ and using the structural formula of ciprofloxacin modified with R substituent, PubMed, Scopus, and Reaxys databases were searched. The articles selected were in English, with free access to the full content, discussing the mechanisms of the action of anticancer drugs, and presenting synthetic protocols for fluoroquinolone derivatives and the results of tests of those compounds for antiproliferative effects on human cancer cells. Furthermore, a snowball approach was used, a result of which the reports relevant to the topic of this article were extracted from the documents cited in this article selected in the main search.

## 7. Conclusions and Future Directions

Cancer pharmacotherapy is constantly being optimised to improve effectiveness and reduce the risk of metastases and complications. Modern scientific reports indicate that the search for new anticancer drugs may be based on the fluoroquinolone pharmacophore system, among other things. The analysis of available data shows that the critical one, from the point of view of the activity of those derivatives, is the *N*-alkylated pyridone system with two carbonyl oxygen atoms: one at position 4 of the pyridone ring and the other in the carboxyl group present at the C3 atom of the pyridone system. Possible modifications in the structure of fluoroquinolone derivatives are primarily applicable to substituents at position 7 of the quinolone system and modifications within the carboxyl group (esterification, amidation). Complexes with metals also seem to be an important direction in the modification of fluoroquinolones to obtain derivatives with anticancer activity. This is confirmed by the complexes of fluoroquinolones obtained so far with Cu(I), Cu(II), Zn(II), Co(II), or Mn(II). It seems that modifications of the fluoroquinolone system at the above-mentioned positions should be taken into account in further research on new anticancer drugs. Attempts to use in silico methods in the design or repositioning of fluoroquinolones as compounds with anticancer activity are also promising.

## Figures and Tables

**Table 1:** Anticancer activity of fluoroquinolones.

Comp. No.	Name and Structure of Fluoroquinolone	Cell Line/Activity	Mechanism of Anticancer Activity	Ref.
**1**	Ciprofloxacin 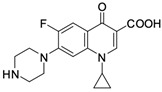	COLO829 melanoma cells/100 µM	Cell cycle arrests in S phase, induction of mitochondrial membrane breakdown leading to apoptosis	[22]
		MDA-MB-231 breast cancer cells/14 µM	Cell cycle arrests in S phase, induction of apoptosis	[23]
		U87MG glioma cells/0.5 µM	Cell cycle arrests in S and sub-G1 phase, DNA fragmentation, and induction of apoptosis	[24]
		LOVO colon cancer cells/110 µM	Induction of apoptosis	[25]
		T24 bladder cancer cells/238 µM	Cell cycle arrests in S phase, induction of apoptosis	[26]
		Huh7/29.4 µM, HepG2/2.9 µM liver cancer cells	Increased expression of CD86+CD206- macrophages leading to inhibition of cell growth	[27]
		Lung cancer cells A549	Cell cycle arrests in G2/M phase	[28]
**2**	Norfloxacin 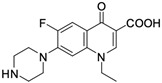	NCI-H460 lung cancer cells/43.5% of inhibition at 200 µM	Antiproliferative effect, generation of apoptosis	[29]
		B16-F10 (mouse melanoma)/<55 µM	Cell cycle arrests in S phase, induction of apoptosis	[26]
		A20 (mouse lymphoma)/<55 µM	Cell cycle arrests in S phase, induction of apoptosis	
**3**	Lomefloxacin 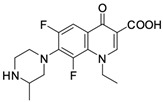	COLO829 melanoma cells/250 µM	Cell cycle arrests in S and G2/M phases, induction of mitochondrial membrane breakdown leading to apoptosis and enhancement of the antiproliferative effect when the drug is exposed to UVA radiation	[30,31]
		HeLa S3 epithelial cancer cells, A431/45.5% of inhibition at 100 µM	Antiproliferative effect, generation of photocleavage of plasmid DNA associated with high photoreactivity of the compound	[32]
		HL-60 leukaemia cells/11% of inhibition at 100 µM	Induction of apoptosis in combination with UVA radiation	[33]
**4**	Levofloxacin 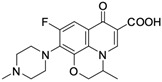	HepG2 hepatocellular carcinoma/60% of inhibition at 250 µM	Cell cycle arrests in G2/M phase and induction of apoptosis	[34]
		MCF-7/58% of inhibition at 5 µM, MDA-MB-231/45% of inhibition at 5 µM, SkBr-2 breast cancer cells/60% of inhibition at 5 µM	Inhibition of cell proliferation, induction of apoptosis	[35]
**5**	Gatifloxacin 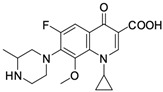	Pancreatic cancer cells MIA PaCa-2/73% of inhibition at 250 µM and Panc-1 pancreatic cancer cells/72% of inhibition at 250 µM	Cell cycle arrests in phases S and G2 without induction of apoptosis	[36]

**Table 3:** IC_50_ values of derivatives 39–48 for MCF-7, A549, and SKOV-3 cell lines.

Compound	IC_50_ [μM]		
	MCF-7	A549	SKOV-3
**39**	3.84	10.24	9.66
**40**	3.58	9.97	7.17
**41**	3.90	6.49	8.50
**42**	3.31	8.52	7.60
**43**	3.26	10.53	5.08
**44**	5.71	14.80	4.14
**45**	3.34	9.69	5.43
**46**	9.48	6.95	3.58
**47**	7.71	5.50	10.57
**48**	15.79	23.51	16.58

## Data Availability

Not applicable.
